# Influenza Virus (H5N1) in Live Bird Markets and Food Markets, Thailand

**DOI:** 10.3201/eid1411.080683

**Published:** 2008-11

**Authors:** Alongkorn Amonsin, Chuensakon Choatrakol, Jiradej Lapkuntod, Rachod Tantilertcharoen, Roongroje Thanawongnuwech, Sanipa Suradhat, Kamol Suwannakarn, Apiradee Theamboonlers, Yong Poovorawan

**Affiliations:** Chulalongkorn University, Pathumwan, Bangkok, Thailand

**Keywords:** H5N1, influenza A virus, live bird market, food market, dispatch

## Abstract

A surveillance program for influenza A viruses (H5N1) was conducted in live bird and food markets in central Thailand during July 2006–August 2007. Twelve subtype H5N1 viruses were isolated. The subtype H5N1 viruses circulating in the markets were genetically related to those that circulated in Thailand during 2004–2005.

In Thailand, from 2004 through 2008, 6 major outbreaks of avian influenza occurred (January–March 2004, July–October 2004, October–December 2005, January–March 2006, November–March 2007, and January 2008). We report on a 14-month avian influenza surveillance program and its finding of influenza virus (H5N1) in live bird and food markets in Thailand.

## The Study

**Figure 1:**
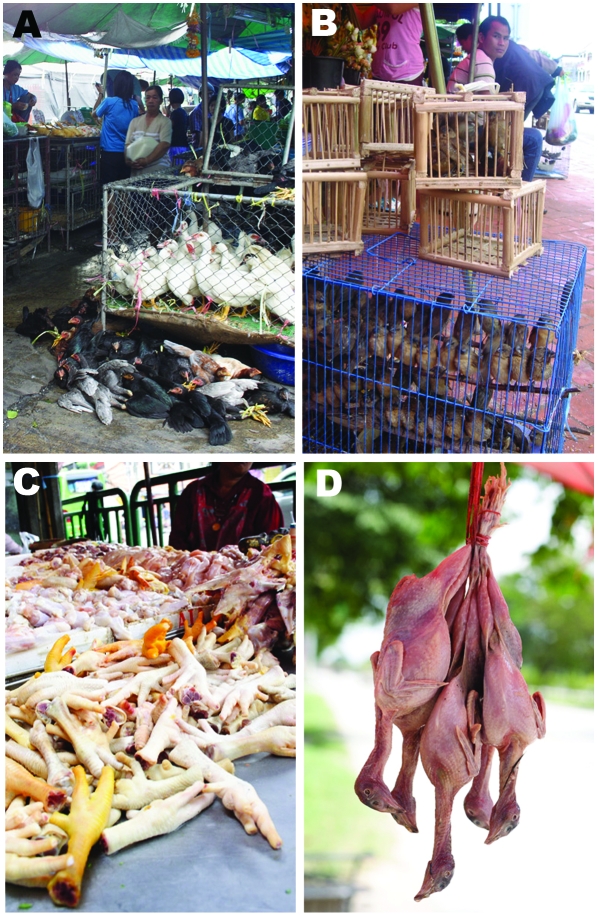
A) Poultry at live bird market; B) house sparrows at live bird market; C) chicken meat at food market; and D) moor hen meat at food market.

From July 2006 through August 2007, an influenza (H5N1) surveillance program was conducted in live bird and food markets in 10 provinces of central Thailand (Figure 1). Cloacal swabs (n *=* 381) were sampled from live chickens, ducks, pigeons, and house sparrows. Visceral organs and bird meats (n *=* 549) were collected from carcasses of chickens, ducks, quails, water cocks, water hens, swamp hens, crakes, parakeets, and moor hens at local food markets (Tables 1, 2). An average of 4 markets (range 1–6) were visited each month, and ≈18 samples were collected from each market. All samples were from backyard animals or local meat birds. None were from birds from standard farming systems with high biosecurity.

**Table 1:** Test results for samples collected during influenza virus (H5N1) surveillance program in live bird and food markets, by collection date, central Thailand

Date sample collected	Location, no. samples		Total no. samples	No. positive results
	Live bird market*	Food market†		
2006				
Jul	7	0	7	–
Aug	6	21	27	–
Oct	8	26	34	–
Nov	20	26	46	5
Dec	18	9	27	5
2007				
Jan	22	35	57	2
Feb	13	36	49	–
Mar	57	66	123	–
Apr	63	74	137	–
May	53	85	138	–
Jun	84	40	124	–
Jul	10	64	74	–
Aug	20	67	87	–
Total	381	549	930	12

*Sample source: swabs. †Sample source: visceral organs and meats.

**Table 2:** Test results for samples collected during the influenza virus (H5N1) surveillance program in live bird and food markets, by bird species, central Thailand

Bird species	Location, no. samples		Total no. samples	No. positive results
	Live bird market*	Food market†		
Chicken	204	3	207	1
Duck	59	2	61	2
Quail	–	396	396	5
Pigeon	6	–	6	–
House sparrow	112	6	118	–
Water cock	–	27	27	2
Water hen	–	33	33	–
Swamp hen	–	1	1	–
Crake	–	1	1	–
Moor hen	–	80	80	2
Total	381	549	930	12

*Sample source: swabs. †Sample source: visceral organs and meats.

The viruses were propagated by embryonated egg inoculation (*1*). Allantoic fluids were tested for influenza subtype H5N1 by hemagglutination (HA). Multiplex reverse transcription–PCR (RT-PCR) was performed to amplify H5, neuraminidase (N) 1, and matrix (M) genes from HA-positive samples (*2*).

Influenza virus (H5N1) was identified in 12 of 930 samples tested. In November 2006, a total of 5 samples with influenza virus (H5N1) were isolated from 1 healthy chicken and 4 visceral organs obtained from 1 live bird market (chicken) and 3 different food markets (moor hen, water cock, and quail). In December 2006, a total of 5 samples with influenza virus (H5N1) were isolated from 5 visceral organs (quail, water cock) from 1 food market. In January 2007, a total of 2 samples with influenza virus (H5N1) were isolated from 2 healthy ducks obtained from 1 live bird market. In the study, 7 isolates were sequenced for whole genome analysis, and the remaining 5 samples were sequenced for H5 and N1 genes. The respective viruses were designated as A/moorhen/Thailand/CU-317/2006 (GenBank accession nos. EU616825–EU616826), A/moorhen/Thailand/CU-318/2006 (EU616827–EU616828), A/watercock/Thailand/CU-319/2006 (EU616829–EU616830), A/quail/Thailand/CU-320/2006 (EU616831–EU616832), A/chicken/Thailand/CU-321/2006 (EU616833–EU616834), A/quail/Thailand/CU-330/2006 (EU616851–EU616858), A/quail/Thailand/CU-331/2006 (EU616859–EU616866), A/quail/Thailand/CU-332/2006 (EU616867–EU616874), A/quail/Thailand/CU-333/2006 (EU616875–EU616882), A/watercock/Thailand/CU-334/2006 (EU61683–EU616890), A/duck/Thailand/CU-328/2007 (EU616835–EU616842), and A/duck/Thailand/CU-329/2007 (EU616843–EU616850).

To analyze the isolates, nucleotide sequences were compared with those of influenza subtype H5N1 viruses in Thailand, People’s Republic of China, Vietnam, Indonesia, Lao, Myanmar, and Cambodia. The sequences were aligned by using the DNASTAR program (*3*) to elucidate and compare the genetic changes. Phylogenetic analysis was conducted by applying the PAUP program (*4*) with the neighbor-joining algorithm and using branch swapping and bootstrap analysis with 1,000 replicates.

## Conclusions

In the course of the 14-month surveillance program, we isolated influenza virus (H5N1) from 12 samples from live birds and from bird meats obtained from the markets. Bird meats were the source of 9 virus-containing samples (5 quail, 2 moor hens, and 2 water cocks), which indicates a risk for influenza virus (H5N1) contamination in bird meats, especially quail. In addition, 3 highly pathogenic avian influenza viruses were isolated from healthy live poultry (1 chicken and 2 ducks). However, the samples that contained influenza virus subtype H5N1 were detected only during the 3-month winter season (November–January). A possible explanation for virus contamination in live bird and food markets may be animal movement from outbreak areas to the markets as well as an attempt to sell infected (dead or dying) birds, especially quail, as bird meat. In addition, most animals or meats in the markets came from backyard farms, where they were in unavoidably close contact with wild birds.

**Figure 2:**
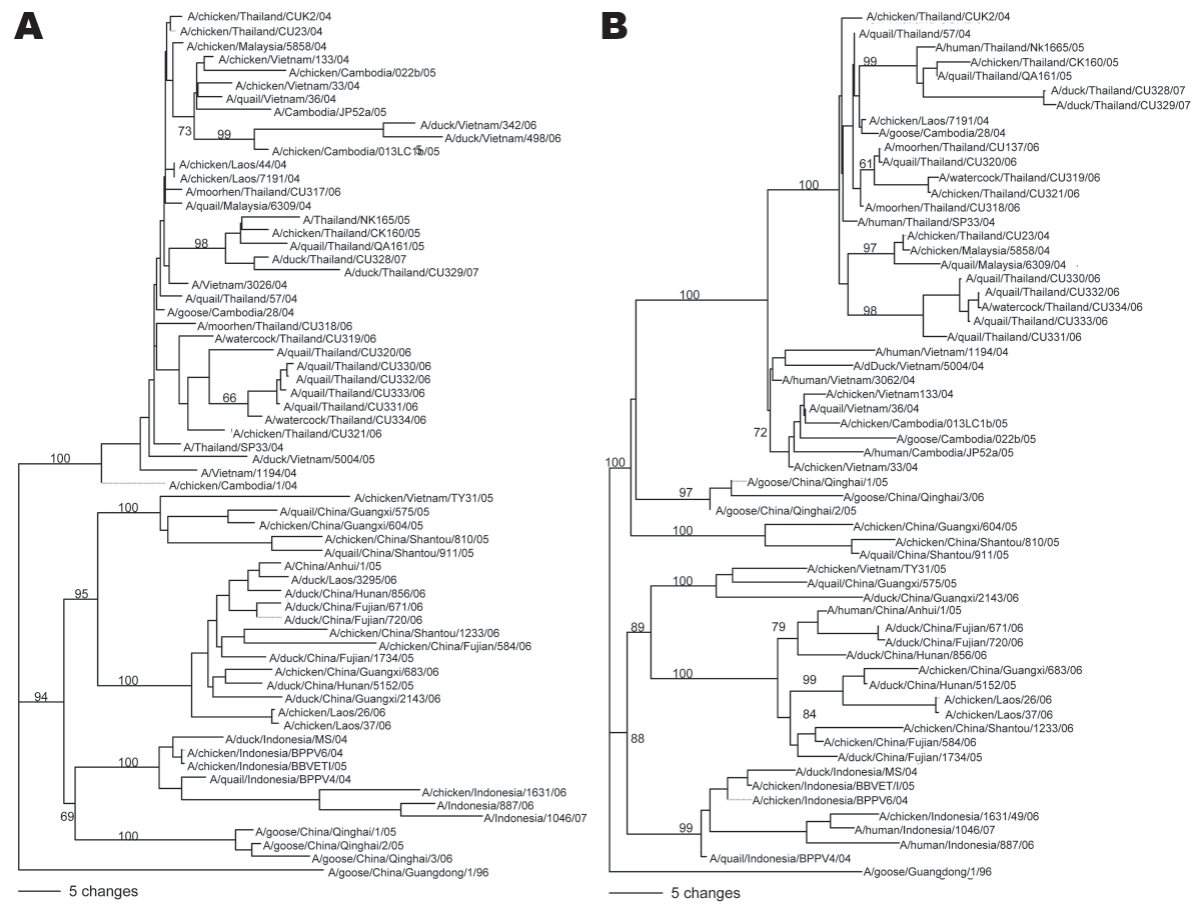
Phylogenetic analysis of the hemagglutinin (A) and neuraminidase genes (B) of influenza virus (H5N1) isolates. Phylogenetic trees were generated by using the PAUP computer program (*4*) and applying the neighbor-joining algorithm with branch swapping and bootstrap analysis with 1,000 replicates. The trees were rooted to A/goose/China/Guangdong/1/96 (H5N1).

Phylogenetic analysis of the virus HA and NA genes indicated that all 12 subtype H5N1 isolates were part of the Vietnam and Thailand lineage (clade 1). The viruses were closely related to those investigated in Thailand (2004–2005) as well as to other subtype H5N1 isolates in clade 1. In contrast, they differed from influenza subtype H5N1 viruses of the south China and Indonesia lineages (clade 2) (Figure 2). In this study, we did not discern any Thailand isolates closely related to the south China lineage, as previously established in Lao and Cambodia (*5*). Phylogenetic analysis of 6 remaining genes showed them to be also closely related to the Vietnam and Thailand isolates.

Analysis of the deduced amino acid sequences of the HA and NA proteins indicated that the viruses had characteristics of highly pathogenic avian influenza. The HA cleavage site consists of multiple basic amino acids RERRRKKR (in 1 isolate, CU-329, REKRRKKR). All influenza subtype viruses harbor Glu-222 and Gly-224 at the receptor binding site, indicating preferential binding to the avian receptor SA-α-2, 3-Gal. In addition, the virus sequences contain 7 glycosylation sites as previously identified in most isolates from Thailand (*6*). A glycosylation site adjacent to receptor binding sites may help increase virus infectivity in host cells (*7*). In some isolates, polymorphisms of amino acids related to antigenic properties of the viruses at position V86A, L138Q, and K140N were observed. All 12 subtype H5N1 viruses had a 20-aa deletion in the NA protein, typical for the NA stalk region of recent subtype H5N1 isolates (2003–2007) (*8**,**9*). None of the subtype H5N1 isolates had any amino acids indicating oseltamivir resistance at the crucial positions 119 (E), 275 (H), 293 (R), and 295 (N) of the NA protein. In summary, the 12 viruses isolated from this study were similar to the viruses from other sources in Thailand, which indicates that the viruses are endemic to Thailand, are circulating in the country, and can be found in any exposed species.

The route of influenza virus (H5N1) introduction into the markets remains to be established. We suspect that this contamination might have occurred as a consequence of animal movement from outbreak areas or from virus-contaminated cages, trucks, and equipment. Unfortunately, the original sources of animals in the markets could not be identified because birds from different sources were housed in 1 or several cages. Fortunately, no human infection was found during 2007–2008 in those provinces where the viruses were isolated.

It has been known that live bird and wet markets play a major role in facilitating emergence or reemergence of influenza and some other respiratory diseases (*10**–**12*). Monitoring of live bird and food markets as an early warning system should be implemented in Asian countries where such markets are still commonplace, and routine surveillance of these markets should be conducted year-round. In addition, raw bird meats should be handled with caution, and consumption of raw bird meats should be avoided. Increased public awareness about the risks for influenza virus (H5N1) in association with live bird and food markets will help prevent and control subtype H5N1 infection in humans.
